# Pathogenesis and multidisciplinary management of medication-related osteonecrosis of the jaw

**DOI:** 10.1038/s41368-020-00093-2

**Published:** 2020-10-21

**Authors:** Lina He, Xiangyu Sun, Zhijie Liu, Yanfen Qiu, Yumei Niu

**Affiliations:** 1 The First Affiliated Hospital of Harbin Medical University, Harbin, China; 2 School of Stomatology, Harbin Medical University, Harbin, China

**Keywords:** Oral diseases, Bisphosphonates

## Abstract

Medication-related osteonecrosis of the jaw (MRONJ) is a serious side effect of bone-modifying agents and inhibits angiogenesis agents. Although the pathogenesis of MRONJ is not entirely clear, multiple factors may be involved in specific microenvironments. The TGF-β1 signalling pathway may have a key role in the development of MRONJ. According to the clinical stage, multiple variables should be considered when selecting the most appropriate treatment. Therefore, the prevention and management of treatment of MRONJ should be conducted in patient-centred multidisciplinary team collaborative networks with oncologists, dentists and dental specialists. This would comprise a closed responsibility treatment loop with all benefits directed to the patient. Thus, in the present review, we aimed to summarise the pathogenesis, risk factors, imaging features, clinical staging, therapeutic methods, prevention and treatment strategies associated with MRONJ, which may provide a reference that can inform preventive strategies and improve the quality of life for patients in the future.

## Introduction

Bisphosphonates (BPs) are the most widely used antiresorptive drugs in the management of cancer-related conditions, such as the prevention of bone metastatic malignancies, and are also used for the treatment and prevention of osteoporosis. However, since the reporting of the first case of osteonecrosis of the jaw (ONJ) in cancer patients who had been treated with high-dose BPs in 2003,^1^ it has been acknowledged that bisphosphonate-related osteonecrosis of the jaw (BRONJ) is a serious adverse reaction to BPs. In response to the incidence of adverse reactions to another bone-modifying agent (BMA), denosumab, or the angiogenesis inhibitor agent bevacizumab, the American Association of Oral and Maxillofacial Surgeons (AAOMS) renamed BRONJ ‘medication-related osteonecrosis of the jaw’ (MRONJ),^2^ as described in a key paper (2014) from the AAOMS.

Drugs that cause MRONJ are grouped into two categories: BPs, used for osteoporosis or malignancy, and non-BPs, including other antiangiogenic or antiresorptive medications. Therapeutic indications, the type of medication and the mode and duration of administration of BPs or antiresorptive therapy are related to the occurrence of MRONJ. The risk of using injectable BPs in patients with malignant tumours is significantly higher when using oral BPs for patients with osteoporosis.^3^ An association with MRONJ has been observed in ~1% to 9% of patients with advanced cancer who are prescribed injectable BMAs.^4^ For patients receiving oral BPs for osteoporosis, the prevalence of ONJ is ~0.21% after at least 4 years of exposure to BPs.^5^

A diagnosis of MRONJ should be considered when patients present with all three of the following criteria: (1) previous or current treatment with a BMA or angiogenesis inhibitor; (2) exposed bone or bone that can be probed through an intraoral or extraoral fistula in the maxillofacial region that has persisted for longer than 8 weeks; and (3) no history of radiation therapy of the jaws or metastatic disease of the jaws.^2^ A pathological diagnosis is helpful for the diagnosis of MRONJ. The most commonly observed pathology is the exposure of bone not covered with epithelium, a reduced number of osteocytes, increased quantities of necrotic bone with a greater number of empty lacunae, demineralised extracellular bone matrix, denudation of the bone and osteonecrosis.^6^ The differential diagnosis of MRONJ should exclude atypical neuralgias, odontalgia, dental caries, pulpitis, periapical pathologies, periodontal diseases, myofascial pain, sinusitis, fibro-osseous lesions, neoplastic processes in the jaw, chronic sclerosing osteomyelitis, alveolar osteitis, sarcomas, or temporomandibular joint disorders. In rare situations, osteoradionecrosis should be strongly considered in patients with an exposed bone who have received treatment with BPs and radiation therapy to the jaw. Inflammation and infections of the bone, with clinical symptoms similar to those of osteomyelitis, are typical secondary events. Furthermore, for patients at risk of or with established MRONJ that have other common clinical conditions, symptoms should not be confused with MRONJ.^7^

## Pathogenesis and risk factors of MRONJ

Although the pathogenesis of MRONJ has not been entirely clarified, several hypotheses have been suggested.

### Unique characteristics of the jaw

Although BPs affect the function of osteoclasts within the skeletal system, only the jaw can suffer from osteonecrosis. The oral cavity has a number of unique characteristics that make it a distinctive environment. The mandible is high in calcium, which may absorb a greater quantity of BPs than the long bones.^8^ Furthermore, the long bones are produced by endochondral ossification, while the mandibles develop principally through intramembrane ossification. Human mandibles have been shown to contain more collagen than long bones.^8^ These anatomical characteristics ensure that the jaw bones are unique, with a propensity to suffer osteonecrosis. The close relationship between the teeth and jaw bone provides a route for microorganisms and other inflammatory agents to enter the bone, a situation not found in any other anatomical location. Dentoalveolar surgery is considered a major risk factor for MRONJ, especially tooth extraction.^9^ The placement of dental implants and endodontic or periodontal surgery requiring exposure and manipulation of bone are also risk factors.

BPs have a greater effect on the cells of the craniofacial bones than those of the ilium and tibia. Mandibular mesenchymal stem cells have demonstrated a higher proliferation rate than long bone mesenchymal stem cells.^10^ It has been reported that the migration of dental stem cells localised in close proximity to the jaw bone decreases following the administration of BPs.^11^ A recent report suggested that BPs can induce the production of reactive oxygen species, which inhibit the proliferation and migration of oral fibroblasts, thereby contributing to the pathogenesis of BRONJ.^12^ Li et al.^13^ found that cell proliferation, adhesion, migration and osteogenic differentiation of periodontal ligament stem cells decreased significantly as a result of BRONJ lesions, a factor possibly important in the underlying mechanisms of BRONJ.

### Altered balance of osteoblasts and osteoclasts in bone remodelling

Bone remodelling is initiated by osteoclast-mediated bone resorption, in which the absorbed bone is replaced by fresh bone tissue produced by osteoblasts. BPs increase osteoclast apoptosis and other antiresorptive drugs inhibit osteoclast differentiation and function, resulting in decreased bone resorption and remodelling. The long bones contain a greater quantity of bone marrow fat than flat bones, and murine long bones contain more osteoclast precursors than jaw bones.^14^ Osteoclasts in the jaw are more sensitive to BPs than those in the long bones.^15^ If the accumulation of BPs within a bone reaches a toxic level, the BPs can affect the survival of osteoblasts and their progenitor cells.

Wehrhan et al.^16^ first demonstrated that TGF-β1 signalling participates in MRONJ. It has an important role in bone remodelling through enhanced matrix production and osteoblast differentiation. Smad 2/3 has been identified as a downstream effector of TGF-β1. A recent study demonstrated that treatment with BPs reduces the expression of BMP-2, which has a major role in bone remodelling, development and osteoblast differentiation. Early differentiation marker type I collagen, intermediate differentiation markers, such as Osterix and alkaline phosphatase (ALP), and the late differentiation marker Osteocalcin has been shown to be suppressed by TGF-β1 combined with low doses of BPs in osteoblasts. In particular, Runx-2 is regulated through Smad 2/3.^17^

Furthermore, TGF-β1 is involved in the synthesis of RANKL through the reduced ability of osteoblasts to secrete RANKL, which stimulates osteoclasts via its receptor, RANK. It has been reported that the treatment of osteoblasts with BPs increases the expression of TGF-β1, resulting in reduced expression of RANKL. OPG, a soluble protein produced by osteoblasts, can inhibit the interaction between RANKL and RANK.^17,18^ BP treatment may alter the RANKL–OPG complex.^17^ The RANK/RANKL/OPG signalling pathway is triggered in MRONJ subjects.^19^ A recent study demonstrated that zoledronate can enhance osteoclastogenesis through elevated expression of interleukin-6 (IL-6), followed by activation of the STAT3 pathway, which is related to vessel regeneration around bone tissues,^20^ and finally the expression of RANKL.^21^ Denosumab, a recently developed antiresorptive medication, is an anti-RANKL antibody that utilises the same mechanism of action as OPG.^22^ By blocking RANKL/RANK interaction, denosumab decreases bone resorption. Using different antiresorptive mechanisms, both BPs and denosumab inhibit osteoclasts and decrease the rate of bone turnover.

Given that the expression of RANKL is altered by multiple signalling pathways, the ratio of osteoblasts to osteoclasts in bone remodelling becomes altered, reducing bone resorption and turnover and giving rise to the accumulation of non-renewed and hypermineralized bone. Changes in the microenvironment of the periosteum cannot provide sufficient nutrition for the jaw, so osteonecrosis occurs following changes in the external environment. Therefore, although bone remodelling representing the pathogenesis of MRONJ may be regulated by multiple signalling pathways, the specific regulatory mechanism should be investigated further.

### Infection and Immunity

Infection or inflammation has long been considered a critical factor in the pathogenesis of ONJ. Bacteria have been found in biopsied specimens of necrotic bone removed from patients with ONJ.^23^ Pre-existing dental or periodontal infection in patients treated with antiresorptive or antiangiogenic medications increases the risk of MRONJ.^24^ Extraction of teeth with serious periodontal or periapical infections is a risk factor for the development of MRONJ.^25^ Another study found that periapical and periodontal infections both with and without tooth extraction can increase the risk of MRONJ because the infection is responsible for modifying the number and function of osteoclasts.^26^ Furthermore, local changes in pH caused by dentoalveolar infection or surgery are the principal factors responsible for the development of BRONJ.^27^ Therefore, inflammatory oral disease is a recognised risk factor for the development of MRONJ. Periodontal or periapical diseases are considered relevant for MRONJ.^28^ Through extensive oral health controls that prevent oral infections, the incidence of MRONJ can be significantly reduced.^7^ The proinflammatory cytokine IL-36 has been found to be present in the gingival crevicular fluid in periodontal diseases. Notably, IL-36α is highly upregulated in MRONJ lesions and has an aetiological role in the development of MRONJ. Importantly, it has been demonstrated that there is crosstalk between the IL-36α and TGF-β signalling pathways,^29^ suggesting that infection or inflammation are key factors in the pathogenesis of MRONJ, at least in part through the TGF-β signalling pathways.

The immune system is closely related to bone loss and bone regeneration. Representing innate lymphocytes, gamma delta T cells are important in bone regeneration. Such T cells are significantly reduced in osteoporotic patients who are treated with BPs, indicating that a connection exists between MRONJ and gamma delta T cell deficiency.^30^ Neutrophils promote wound healing following noninfective injury. Nitrogen-containing BPs alter the defence capabilities of neutrophils and impair normal wound healing, possibly representing a critical role in the pathogenesis of MRONJ.^31^ Macrophages are sensitive to BPs, which cause an inhibitory effect and reduce the viability and differentiation capability of the macrophages. The function of macrophages is disrupted by increased MMP expression, leading to impaired wound healing in MRONJ-affected areas.^32^ Through the inhibition of RANKL, denosumab may affect the expression of RANK on immune cells, such as dendritic cells, monocytes, or macrophages. RANKL increases the production of proinflammatory cytokines and reduces monocyte apoptosis. Thus, denosumab inhibits the RANK–RANKL interaction, resulting in MRONJ, which may be related to a change in the function and survival of monocytes and macrophages.^33^ Therefore, as weak evidence, both BPs and denosumab might facilitate infection of the bone surface, resulting in an increased risk of MRONJ. Interleukins, proteins produced by immune cells, are related to the expression and regulation of the immune response, which is involved in multiple factors from lymphocytes and macrophages. It is noteworthy that IL-6 and IL-36α expression are elevated following treatment with BPs.^29^ IL-6 subsequently activates the STAT3 pathway, while IL-36α activates the ERK signalling pathway and subsequently inhibits translocation of TGF-β1 and the Smad signalling pathway.^21,29^ Furthermore, TLR-4-mediated macrophage polarisation participates in the pathogenesis of BRONJ in mice.^34^ Therefore, it is possible that multiple signalling pathways participate in the pathogenesis of MRONJ.

### Angiogenesis

Vascular endothelial growth may be a critical factor in the pathogenesis of MRONJ.^35^ Zoledronate has direct inhibitory effects on angiogenesis and vascular damage, possibly contributing to the development of MRONJ in its users, owing to reduced angiogenesis impairing healing after the intervention.^36^ The antiangiogenic effects of denosumab and zoledronate were compared, and the findings suggest that zoledronate exhibits negative effects on angiogenesis, while denosumab may not have antiangiogenic activity.^37^ Vascular endothelial growth factor (VEGF) has an essential role in angiogenesis. The antiangiogenic properties of BPs are directly linked to the pathogenesis of MRONJ, and serum VEGF levels could represent an effective early predictive marker.^35^ Monoclonal antibodies targeting VEGF receptors,^38^ as antiangiogenic drugs, are prescribed in cancer patients to prevent metastasis through the blood and lymph nodes, resulting in ischaemia and eventually MRONJ.^39^ VEGF synthesis is stimulated by TGF‑β.^40^ The expression of TGF-β and angiogenesis-related signalling have been shown to be possible consequences of MRONJ.^41^ Altered VEGF expression has been observed following treatment with BPs,^42^ possibly related to the expression of TGF-β.^16^

### Soft tissue toxicity

Although osteoclasts and bone are the primary targets following their exposure to BPs, it has been reported that the toxicity of BPs to soft tissue is closely related to MRONJ. Mucosal ulcerations may be the initial pathologic event that occurs in MRONJ.^43^ BPs increase apoptosis and decrease proliferation in a number of cell types in vitro.^12,13^ In addition, the administration of zoledronic acid to oral gingival fibroblasts in vitro has been found to reduce the expression of extracellular matrix (ECM) proteins, including collagens I, II and III.^44^ It has been shown that impairment of TGF-β1 signalling is related to oral mucosal soft tissue repair in BRONJ.^16^ Increased TGF-β1 and Smad 2/3 expression are related to fibrocontractive wound healing disorders.^45^ Alterations in TGF-β1 signalling after BP treatment might explain BP-associated changes in the oral mucosal tissues of MRONJ.

### Other factors

Systemic diseases may increase the risk of MRONJ. BPs are occasionally administered to patients with rheumatoid arthritis to reduce bone destruction and control osteoporosis.^46^ However, BPs have been found to be associated with MRONJ in such patients, especially following their use over long durations and in high doses. Administration of zoledronate, among the most common BPs used clinically, has been shown to result in more serious MRONJ in experimental mice with rheumatoid arthritis.^47^ Therefore, rheumatoid arthritis may be a risk factor for the pathogenesis of MRONJ. Diabetes mellitus promotes inflammation and induces a change in the function of immune cells, which may affect the pathogenesis of MRONJ. It has been reported that diabetic mice are more likely to suffer from MRONJ. The relationship between diabetes mellitus and the pathogenesis of MRONJ is also related to other pathways of injury, such as microvascular ischaemia and reduced bone remodelling.^48^

Genetic factors have a moderate effect on the occurrence of MRONJ. There is an association between the presence of one or more single nucleotide polymorphisms (SNPs) and the appearance of MRONJ.^49^ The majority of SNPs are located in regions of genes associated with bone turnover or certain metabolic bone diseases. In addition, there may be germline sensitivity to BPs.^50^ Corticosteroids also increase the risk of MRONJ.^9^ Age, gender, tobacco use and type of cancer are variable risk factors for MRONJ.^51–53^

**Fig. 1:**
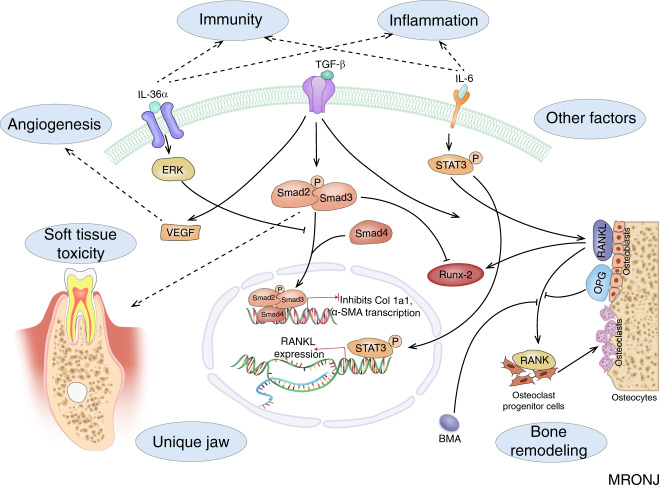
The hypotheses of MRONJ pathogenesis.

Although the hypotheses above have been developed to elucidate the pathogenesis of and risk factors for MRONJ, the mechanisms are not yet entirely clear. TGF-β1 signalling, a relatively well-elucidated pathway closely related to the pathogenesis of MRONJ, may have a key role in the development of MRONJ (Fig. 1). Additional mechanisms underlying the pathophysiology of MRONJ remain to be elucidated.

## Clinical staging and imaging features of MRONJ

**Fig. 2:**
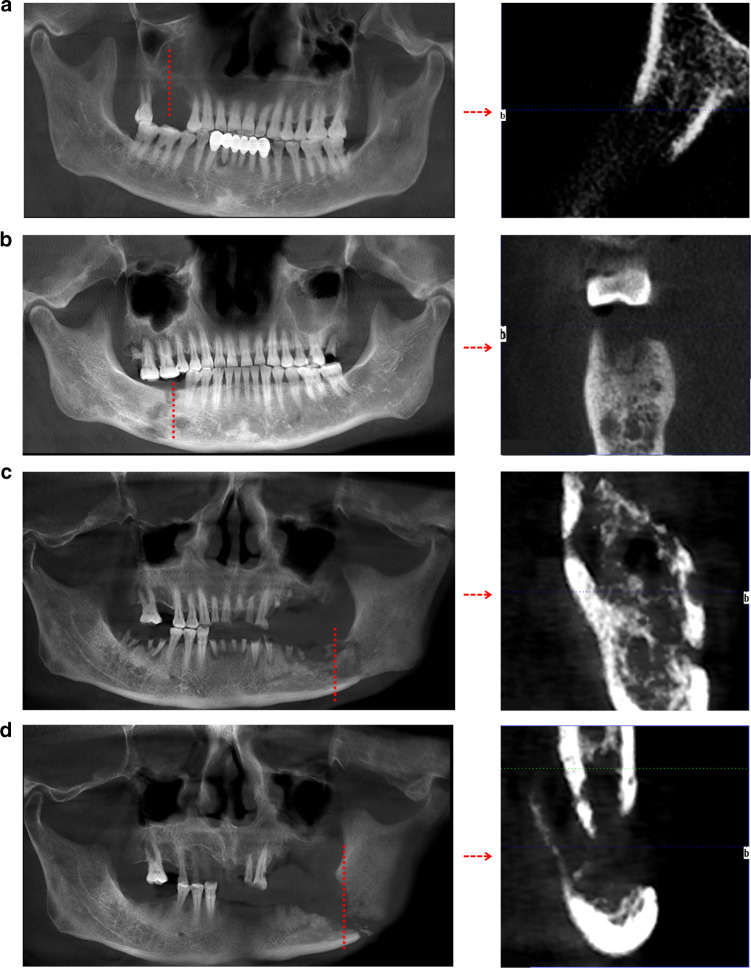
Imaging findings in different clinical stages.

A clinical staging system was used to categorise patients with MRONJ. Patients with no apparent necrotic bone are considered to be ‘at-risk’ if they have been treated with bone-modifying agents. Stage 0 patients have no clinical evidence of necrotic bone but present with nonspecific symptoms or clinical and radiographic findings. Stage 1 is defined as patients having exposed and necrotic bone or fistulas that probe to the bone and who are asymptomatic and have no evidence of infection. Stage 2 is defined as individuals with exposed and necrotic bone and who have pain and clinical evidence of infection. Stage 3 is defined as patients with exposed and necrotic bone or fistulas that probe to bone with evidence of infection and at least one defined characteristic. According to the AAOMS, imaging is critical in the diagnosis and assessment of disease progression in MRONJ patients.^27^ Orthopantomography, CT scanning and MRI are most commonly used to diagnose MRONJ. A recent study demonstrated that the qualitative assessment of MRONJ with ultrashort echo-time (UTE) magnetic resonance (MR) imaging was comparable to the reference standard cone-beam computed tomography (CBCT).^54^ Bone scintigraphy contributes to the early detection of MRONJ in high-risk patients.^55^ In animals with MRONJ, the most consistent macroscopic findings are necrosis, denuded bone and formation of fistula and pus.^6^ Arce et al. assessed patients with MRONJ using six techniques, including films and magnetic resonance imaging. The imaging findings included osteosclerosis, osteolysis, dense woven bone, thickened lamina dura, subperiosteal bone deposition and failure of postsurgical remodelling.^56^ Imaging findings are different for different clinical stages (Table 1 and Fig. 2), but the definition of MRONJ does not currently include imaging-related criteria.^4^ Compared with typical imaging changes in stages 2 and 3, such as large-scale necrotic bone separation or pathological fracture, more attention should be paid to changes observed in the imaging of patients at stage 0. The capture of subtle changes in bone (as shown in Fig. 2a), which are not easy to detect, may have an important role in the follow-up treatment to delay the progression of the disease.

**Table 1:** Clinical conditions and Imaging features by stage of MRONJ according to the American Association of Oral and Maxillofacial Surgeons

MRONJ stage	Clinical conditions	Imaging features
At risk	No apparent necrotic bone in patients treated with bone-modifying agents	Nonspecific radiographic changes
Stage 0	No clinical evidence of necrotic bone, but nonspecific clinical findings and symptoms	Alveolar bone loss or resorption Clerotic alveolar bone, thickening and sclerosis of the lamina dura Thickening or obscuring of the periodontal ligament
Stage 1	Exposed and necrotic bone or fistulas that probe to the bone in patients who are asymptomatic and have no evidence of infection	May present same as stage 0 Changes to trabecular pattern: disorganised, trabecular pattern and poor corticomedullary differentiation
Stage 2	Exposed and necrotic bone in patients with pain and clinical evidence of infection	Mixed diffuse osteosclerosis, osteolysis from the alveolar bone to the jaw bone, thickening of the mandibular canal, periosteal response, maxillary sinusitis and sequestration
Stage 3	Exposed and necrotic bone or a fistula that probes to the bone in patients with pain, infection and one or more of the following: exposed and necrotic bone extending beyond the region of alveolar bone resulting in pathologic fracture, extraoral fistula, oral antral or oral-nasal communication or osteolysis extending to the inferior border of the mandible or sinus floor	Osteosclerosis/osteolysis of the surrounding bone, pathologic mandibular fracture and osteolysis extending to the maxillary sinus floor

## Prevention and MDT treatment strategies for MRONJ

Multiple variables, including age, sex, disease status, MRONJ stage and lesion size,^7^ should be considered prior to the assessment of risk and selection of a treatment plan. Therefore, personalised treatment plans and multidisciplinary collaborative therapy should be used clinically. The collaboration of oncologists, dentists and dental specialists should be practised at every period of treatment, sharing treatment information contemporaneously.

The key to the prevention and treatment of MRONJ is its detection and diagnosis at an early stage to avoid the risk of progression and effectively prevent its occurrence by screening high-risk patients who are prone to the disease (e.g. AAOMS stage 0).^4^ Therefore, prevention should precede treatment. Published studies have suggested that oral hygiene and treatment of local infections can reduce the risk of MRONJ.^57–59^ Close follow-up and multidisciplinary collaborations between dentists and oncologists have a vital role in the prevention and treatment of MRONJ. A multidisciplinary collaboration system should be established.

More attention should be paid to oral care and periodontal and general health education for all stages of MRONJ.^60^ Treatment strategies for the category include the treatment of patients with modifiable risk factors and the management of avoidable risk factors. Maintenance of meticulous oral hygiene and the use of antibacterial mouth rinses can help delay the progression of MRONJ.^4^

Cancer patients who plan to receive BMA in non-emergency situations should be evaluated for oral care prior to the start of treatment to ensure that the necessary medical dental procedures are performed before the start of BMA.^4^ Dental follow-up should be conducted using a routine schedule, such as every 6 months following the start of BMA treatment,^7,61^ but a clear plan for monitoring the progress of the disease has not been put forward in detail.^62^ Multidisciplinary team members should work with patients at an early stage to address the risk factors for MRONJ. Selective alveolar procedures should not be performed during aggressive treatment with BMA at tumour doses.^63^

For prevention and the process management of MRONJ, multidisciplinary experts and patients themselves should be involved to improve the process,^4^ performing their individual roles (Table 2). Oncologists should inform patients about the importance of oral care prior to treatment and that they should undergo an examination by a dentist to eliminate, as far as possible, any risks. For patients recently diagnosed with MRONJ, whether BMA therapy should be continued or discontinued can be ascertained, determining the development of subsequent treatment plans. Modifiable risk factors should be reinforced: invasive dental procedures, diabetes, periodontal disease, denture use and smoking. The dentist should also be provided with information about the patient’s medical diagnosis and antiabsorption and angiogenesis inhibitors used to indicate whether the patient had already commenced therapy and its duration.^64^

**Table 2:** Division of tasks for multidisciplinary management of MRONJ

Participating disciplines	Task division
Oncology	1. Determine the continuation or discontinuation of BMA therapy in patients 2. Refer patient for dental assessments and need for commitment to oral care 3. Reinforce modifiable risk factors 4. Provide the dentist with the patients’ medical diagnosis and antiresorptive and angiogenic inhibitor profile 5. Indicate if the patient has already commenced therapy and duration
Dentist	1. Receive patient, evaluate modifiable risk factors, establish follow-up system 2. Before antiresorptive therapy: • Conduct complete dental examination • Perform necessary dental extractions and conservative dental and periodontal interventions • Adjust prosthetics • Educate the patient about the need for a lifelong daily commitment to oral care • Encourage the correction of risk factors 3. During antiresorptive therapy: • Encourage follow-up visits every 6 months • Conduct complete dental examination • Evaluate the oral status of oral soft and hard tissue • Reinforce ongoing education about the importance of maintaining good oral hygiene • Continue to reinforce modifiable risk factors 4. Follow patient’s lesion status and report it to the oncologist
Dental specialist	1. Accept suspected patients 2. Management is determined by the stage, the severity of symptoms, functional impact and overall prognosis and should be on an 8-week follow-up schedule 3. Design a treatment plan and inform oncologist 4. Evaluate disease outcome 5. Make sure follow-up visits every 8 weeks

For dentists, treatments should be consistent, with a follow-up system established that ensures follow-up examinations. For patients prior to cancer treatment, modifiable risk factors should be evaluated, and the required pre-treatments should be improved: complete dental examinations, including orthopaedic photography and intraoral radiography, required extractions, conservative dental and periodontal treatments, adjustment of prosthetics if required, and finally education about the requirement for a lifelong daily commitment to oral care and encouraging the correction of risk factors (such as smoking^65^ and uncontrolled diabetes^66,67^). Controllable risk factors should be minimised. During the course of treatment, follow-up for 6 months should be strictly adhered to, and the doctor should be consulted at any time if symptoms reappear. It is recommended that the dentist complete an oral examination, evaluate the status of the soft and hard tissues in the oral cavity, continue oral education and control risk factors. Patients should ensure that they communicate with their oncologist at any time.^68–70^

When a suspected MRONJ patient consults a dentist or oncologist, the patient should be referred to a dental specialist for additional treatment management.^71^ The dental specialist should evaluate the clinical stage, the severity of the disease and symptoms, functional impact and overall prognosis based on clinical manifestations following an 8-week follow-up plan. In accordance with the staging of MRONJ, the dental specialist should design a treatment plan for its management and report that plan to the oncologist. The patient should be followed-up for 8 weeks, the outcome of which should be assessed by a dental specialist.^72,73^

Prevention and treatment management of MRONJ should be patient-centred through an MDT collaborative network with oncologists, dentists and dental specialists. Each treatment team should be responsible for each stage of the patient’s progress to complete the corresponding tasks (Table 3). The treatment team also needs to share treatment resources and be able to communicate at any time to develop a follow-up treatment plan.^74^ For the patient, this would be a treatment loop of closed responsibility with all benefits directed to the patient. This proposed model represents a new paradigm for the management of MRONJ that is suitable for widespread promotion.

**Table 3:** Multidisciplinary cooperation mode and management strategy by stage of MRONJ

MRONJ stage	Treatment strategies	Participating disciplines	Management strategies
At risk	Patient education Maintain meticulous oral hygiene	Oncology	Reinforce modifiable risk factors
		Dentist	Conduct complete dental examination Encourage the correction of risk factors Establish follow-up system
Stage 0	Oral antibacterial mouth rinse Perform medical treatment (antiseptic, analgesic, antibiotic therapy etc.) Low-intensity laser therapy	Dentist	Encourage follow-up visits Evaluate oral status of oral soft and hard tissue Follow patient’s lesion status and report it to the oncologist
		Oncology	System management, including the use of pain drugs and antibiotics
Stage 1	Use antiseptic fluids to rinse the exposed/necrotic bone and fistulae Low-intensity laser therapy Perform medical treatment 8-week follow-up to decide the further treatment plan	Dentist	Clinical follow-up Patient education Detailed examination of oral state Application of antibacterial mouthwash
		Dental specialist	Receive patient, make 8-week follow-up plan Evaluate basic information of patients
		Oncology	Patient education and review of indications for continued BP use based on stage 0
Stage 2	Low-intensity laser therapy Perform further medical treatment (Teriparatide, Pentoxifylline and Tocopherol etc.) Conservative surgical to remove all affected bone to minimise inflammation Adjuvant surgical therapy (PRP, hyperbaric oxygen etc.) Soft tissue defect management	Dentist	The same as stage 1 Evaluate the soft and hard tissues in the oral cavity
		Dental specialist	The same as stage 1, if infection is suspected Conduct symptomatic treatment Use systemic antibiotics Consider surgical debridement Alleviate the symptoms of patients
		Oncology	Perform pain management Focus on patients’ systemic factors and the development of basic diseases after discontinuation of drugs
Stage 3	Continue the treatment strategy of stage 2 to slow down the progress of disease Radical invasive surgery (when conservative treatment is ineffective) Defect reconstruction with free flap	Dentist	The same as stage 1 and 2 Emphasis on the application of antibiotic mouthwash and the education of maintaining good oral hygiene
		Dental specialist	The same as stage 1 and stage 2 Perform surgical debridement and resection when conservative treatment is ineffective Follow patient’s lesion status and report it to the oncologist Lesion status: • Resolved: complete healing • Improving: significant improvement (>50% of mucosal coverage) • Stable: mild improvement (<50% of mucosal coverage). • Progressive: no improvement.
		Oncology	The same as stage 1 and 2 Pay attention to pain management and improve the quality of life of patients Determine the strategy of drug follow-up application

### Therapy for MRONJ

From an in-depth study of MRONJ, conservative therapy is the mainstream treatment,^75–77^ as it can provide long-term relief.^9^ According to the Clinical Practice Guidelines of the MASCC/ISOO/ASCO, for patients with confirmed MRONJ, the treatment goal is to alleviate pain, control infection in soft and hard tissues, and decrease the progress or occurrence of osteonecrosis.^4^ This may be interpreted as the fact that the treatment for MRONJ has been clearly defined, delaying the progress of the disease with minimal cost to provide patients with a better after-treatment experience. Studies have reported that there is no significant difference in the rate of achieving a cure between surgical and nonsurgical treatments,^78,79^ with less aggressive surgical treatments producing better outcomes than those that are more aggressive.^80^ Therefore, in clinically asymptomatic conditions, conservative treatment would be the primary choice. New guidelines state that it is not recommended to intervene in asymptomatic bone exposure with aggressive surgical treatment. Prior to treatment, the multidisciplinary team should discuss thoroughly with the patient the risks and benefits of the proposed plan.^4^

Reported cases of successful treatments of MRONJ using teriparatide (TPTD) have verified that it is beneficial for osteoporotic patients with established MRONJ. TPTD has been used to treat MRONJ in animal studies, and positive outcomes have been observed.^81^ Successful treatment with TPTD for MRONJ has been found in several clinical case reports.^82,83^ Combination treatment with bone morphogenetic protein can enhance bone formation and promote bone regeneration.^84^ In patients with stage 3 MRONJ refractory to conservative management, weekly TPTD administration was shown to be as effective as daily TPTD in promoting bone healing and removing osteonecrotic tissue.^85^ However, no additional prospective randomised studies with convincing results of the treatment of MRONJ with TPTD have been published, so it is too early to support the use of TPTD as prevention or treatment for MRONJ.^86^

The combination of pentoxifylline and tocopherol has been used previously in the management of osteoradionecrosis, with significant improvement in symptomatology.^87^ Pentoxifylline is a nonselective phosphodiesterase inhibitor that displays good therapeutic results against osteoradionecrosis of the jaws and MRONJ.^88–91^ Tocopherol is a powerful scavenger of oxygen free radicals, which can reduce the damage caused by free radicals and necrosis.^92^ The study demonstrated that pentoxifylline and tocopherol are relatively inexpensive and simple to use and represent a safe and effective treatment for MRONJ.^87^ Studies have reported that pentoxifylline and tocopherol are potentially useful for the nonsurgical management of MRONJ.^88,89^ This treatment provides similar results towards healing as other nonsurgical treatment modalities with minimal side effects, costs and/or time burdens. Additional studies are required, however, to determine the optimal dosing and duration of treatment.

Antibiotics should not be abused at any stage of MRONJ^93^ because infection does not directly lead to its development. However, the majority of MRONJ patients have infection-related symptoms (stages 2 and 3). Therefore, at stages 2 and 3 with a substantial area of necrosis, antibacterial therapy contributes to the healing of MRONJ. When conservative treatment is unsatisfactory, surgical treatment is required.^94,95^ Surgical treatment modalities vary from marginal osteotomy to segmental osteotomy. Following the failure of conservative treatment, surgery is widely recommended in patients with hypoimmunity and reduced quality of life to avoid the risk of bacteraemia and septicaemia.^96–98^ It has been reported that weak patients with MRONJ who are unresponsive to conservative methods may have oral complications, including exposure to bone, infection, pain and discomfort, therefore requiring specialised care.^99–101^ Considering that these circumstances promote infection, it is necessary to perform surgery therapeutically.^102^ However, there is no clear guidance on how to determine the surgical plan for patients in stages 2 and 3 for whom conservative treatment was ineffective, so surgical treatment is therefore advocated.^103–105^ However, the type of recommended surgical intervention, including preservation or segmental resection of the jaw, determination of the incisal margin of safety, soft tissue management and selection of functional reconstruction, is not clear.

Because the surgical treatment of MRONJ is difficult to quantify, expansion of the range of surgical resection is an important but uncertain factor for establishing the success of surgical treatment.^103,106^ The boundary for marginal osteotomy is based on the findings from the operation, such as bleeding and colour of the remaining bone, which are not always positively correlated with bone vitality. Inexperienced maxillofacial surgeons do not always find that performing marginal resection surgery is simple. It has been reported that bone resection could be performed under fluorescence guidance, and necrotic osteotomy selected during surgery could assist in distinguishing necrotic bone from healthy bone for standardising surgical treatment.^107^ Experienced maxillofacial surgeons are believed to have the capability to successfully treat affected areas using osteotomy.

There are also challenges in the restoration of soft and hard tissue function following the resection of a lesion. Due to soft tissue toxicity, unlike free flap reconstruction, buccal fat pad (BFP) flaps have been used to fill the dead space in necrotic bone resection.^108,109^ The main advantage of BFP flaps is their rich blood supply, flexibility, lack of age restrictions, safety and spontaneous formation of the epithelium.^110,111^ It has been reported that the use of BFP flaps makes it possible to close maxilla defects with two layers (BFP and mucoperiosteal flaps).^112^ Using this method, the majority of bone surfaces can be closed with BFP flaps, avoiding oral and nasal communication and infections of the maxillary sinus. Undoubtedly, this is the most appropriate treatment selection. Even if the BFP flap becomes exposed, spontaneous epithelialization occurs, which results in defect reconstruction.

Hyperbaric oxygen therapy is an effective adjuvant treatment for MRONJ. Hyperbaric oxygen can reduce inflammation and oedema and increase vasculogenesis, antimicrobial activity and tissue repair.^113,114^ It has been reported that hyperbaric oxygen alone cannot completely cure MRONJ.^115^ Therefore, it should be considered part of multimodal therapy. Hyperbaric oxygen is beneficial when used with antibiotics and surgery.^116,117^ Although current findings demonstrate that hyperbaric oxygen can improve MRONJ, the methodological limitations contribute to a lack of measurable effects.^118,119^ Therefore, the efficacy of hyperbaric oxygen therapy in MRONJ treatment requires further investigation.

Owing to the beneficial effects of low-intensity laser (LIL) treatment towards tissue healing, both alone and in combination with other treatment methods such as high-power lasers and surgical intervention, improved therapy can be achieved^120–122^ because LIL treatment can regulate metabolism, promote wound healing and relieve pain.^123^ It has been established that biological stimulation from LILs assists in healing both soft and hard tissues, especially in the treatment of early pathological changes.^124,125^ When combined with a high-power laser, a LIL is effective in inducing the complete healing of mucosa and reduction in microbial contamination. Studies have shown that surgery is an important treatment for patients with MRONJ, but when a LIL is used together with the removal of necrotic bone, better therapeutic effects can be obtained.^123,124^ Therefore, LIL has become an effective adjuvant therapy for MRONJ and is expected to become an alternative therapy.^104^

Platelet-rich plasma (PRP), an autologous source of growth factors, has been successfully used in bone regeneration and soft tissue healing.^126,127^ PRP can produce high concentrations of human platelets containing a variety of growth factors. In surgery for MRONJ, PRP appears to be particularly useful as an adjunct to surgical debridement and marginal osteotomy in patients with conservative treatment failure.^128,129^ However, there is no clear support for current PRP treatments, and more research is required to assess the potential of the treatment.^130,131^

Following an in-depth study of the disease, multiple therapeutic methods have been proposed, which can affect the outcome of the disease to a certain extent. There is no clear guidance on how to standardise the choice of treatment method from the multitude of effective alternatives. A sequential treatment pattern should be proposed, as shown in Table 3. The management and mode of treatment cooperation in multidisciplinary teams (MDTs) should be conducted during all periods of treatment. At all stages of disease progression, patient education should be emphasised and oral hygiene should be controlled by simple methods, such as oral antibacterial mouth rinse, which has an important role in inhibiting the development of the disease. LIL therapy is a possible choice for the treatment of osteonecrosis by aiding the reparative process. LILs can stimulate the growth of the vascular system and blood capillaries.^123^ At stage 0 in particular, the use of LIL therapy can control the progress of the disease, consistent with the treatment concept. At stage 1, the prevention of progressive infection is the key to blocking disease progression. The use of local antiseptic fluids to rinse the exposed/necrotic bone and fistulae and the application of LIL can provide a better local healing environment, promoting the prognosis of osteonecrosis. An 8-week follow-up examination should be performed, and a multidisciplinary consultation should determine the follow-up treatment plan based on the follow-up results. At stages 0 and 1, the principal purpose of drug treatment is to prevent infection and control symptoms. When the disease progresses to stages 2 and 3, drugs such as teriparatide, pentoxifylline and tocopherol can be used to delay the development of the disease, and the progress of infection can be controlled using antibiotics. When a drug or auxiliary LIL treatment for more than 2 weeks has no apparent effect, conservative surgery should be considered to remove all affected bone to minimise inflammation. At the same time, auxiliary treatment such as PRP or hyperbaric oxygen can be used to increase the success rate of surgery.^104,132^ After bone tissue infection has been controlled, an adjacent flap with abundant blood supply (such as a BFP or the modified submental island flap) should be first considered for the repair of soft tissue defects.^133^ In stage 3, in case of ineffective conservative treatment, radical invasive surgery can be conducted to improve the quality of life of the patient. However, in order to reconstruct the dead space following the removal of damaged bone, free flap repair can be used to reconstruct soft tissue defects, which should be performed prior to correcting the hard tissue defect. From the summary of existing and effective treatment methods, it is hoped that standardised sequential therapy can be formulated as soon as possible.

## Prospect

The pathogenesis of MRONJ has not been entirely clarified and may involve many factors within specific microenvironments. Multiple signalling pathways may be involved in the pathogenesis of MRONJ. At present, it is relatively certain that the TGF-β1 signalling pathway has a key role in the development of MRONJ,^16,29,41^ potentially representing a future research direction. Therefore, the study of the TGF-β pathway and specific downstream sites may lead to a breakthrough in the pathogenesis of MRONJ. Although there are many studies on MRONJ, a consensus on a sequential treatment plan still requires additional research. If the accumulation of BPs in the jaw can be competitively inhibited while maintaining a drug-based treatment, this may be an effective method of preventing osteonecrosis of the jaw in the future. The multidisciplinary collaboration model of disease management should be affirmed. During treatment, each team member should integrate and share information about the treatment and perform his or her specified role at each stage, thereby preventing the occurrence or development of MRONJ to accomplish personalised treatment.
